# Optimizing Resource Utilization in Low- and Middle-Income Country NICUs: A Clinical Audit of Surgical Infection Screening Practices at a High-Volume NICU in Pakistan

**DOI:** 10.12669/pjms.42.(11AASC).15734

**Published:** 2026-04

**Authors:** Humza Thobani, Rafia Durrani, Muhammad Osama Khan, Javeria Javed, Sulaiman Sajjad, Zahra Iftikhar, Muhammad Aqil Soomro, Saqib Qazi, Faraz Ali Khan, Saleem Islam

**Affiliations:** 1 Dr. Humza Thobani, MBBS. Section of Pediatric Surgery, Department of Surgery, Aga Khan University, Karachi, Pakistan. Division of Pediatric Surgery, Department of Surgery, Stanford University, Palo Alto CA; 2 Dr. Rafia Durrani, MBBS, Section of Pediatric Surgery, Department of Surgery, Aga Khan University, Karachi, Pakistan; 3 Dr. Muhammad Osama Khan, MBBS. Section of Pediatric Surgery, Department of Surgery, Aga Khan University, Karachi, Pakistan; 4 Dr. Javeria Javed, MBBS. Section of Pediatric Surgery, Department of Surgery, Aga Khan University, Karachi, Pakistan; 5 Sulaiman Sajjad, School of Medicine, Imperial College of London, United Kingdom; 6 Dr. Zahra Iftikhar, MBBS. Section of Pediatric Surgery, Department of Surgery, Aga Khan University, Karachi, Pakistan; 7 Dr. Muhammad Aqil Soomro, FCPS. Section of Pediatric Surgery, Department of Surgery, Aga Khan University, Karachi, Pakistan; 8 Dr. Saqib Qazi, FCPS. Section of Pediatric Surgery, Department of Surgery, Aga Khan University, Karachi, Pakistan; 9 Dr. Faraz Ali Khan, MD. Division of Pediatric Surgery, Department of Surgery, Stanford University, Palo Alto CA; 10 Dr. Saleem Islam, MD, MPH. Section of Pediatric Surgery, Department of Surgery, Aga Khan University, Karachi, Pakistan

**Keywords:** Neonates, Surgical infections, CRP, Blood culture, NICU

## Abstract

**Objective:**

Post operative sepsis in neonates is a serious problem that may be challenging to diagnose. It is standard practice at our Neonatal Intensive Care Unit (NICU) in Pakistan to perform routine Blood Cultures (BLCS) and C-Reactive Protein (CRP) to screen for post-operative sepsis. We aimed to review this practice to investigate its effectiveness at screening for post-operative sepsis.

**Methodology:**

All neonates admitted to the NICU post-operatively at our center from 2017-2022 were included. Relevant clinical and demographic data were collected. The sensitivity of BLCS was calculated for each post-operative day (POD) and an ROC curve was constructed for overall CRP values to quantify their screening value.

**Results:**

A total of 109 post-operative neonates were included (median gestational age 37 weeks, birth weight 2.4kg). Thirteen (12.6%) developed sepsis. Only two patients had pathological microbe growth on POD 0 or 1, both having growth preoperatively. BLCS sensitivity increased significantly after POD 2. CRP performed poorly at discriminating post-operative sepsis (AUROC=0.55).

**Conclusion:**

Routine BLCS performed immediately after surgery did not predict the onset of post-operative sepsis. CRP performed poorly at discriminating post-operative sepsis, likely due to physiologic inflammation in post-operative neonates. Unnecessary screening tests represent a significant financial burden in LMICs, with little clear clinical benefit.

## INTRODUCTION

Caring for patients with neonatal surgical disease is very challenging in Low- and Low- to Middle-Income Countries (LICs, LMICs), where outcomes are significantly poorer than in High-Income Countries, given in specialized tertiary care settings.^1^-^3^ Providing neonatal care in resource-limited settings brings enormous challenges, as this is often guided by not just the health issues, but by financial considerations to the health system and the families. Quality improvement (QI) programs in LMICs to improve neonatal surgical outcomes thus have the added tasks of identifying and reducing unnecessary expenditures in addition to optimizing outcomes. Unfortunately, QI initiatives are underutilized in LMICs, leading to care that is often inefficacious, uneconomical and ultimately bad for patients and families alike – many of whom experience catastrophic health expenditure while trying to decide on saving their critically ill children, a Hobbesian choice.^4^

Post-operative infections and sepsis in particular contribute significantly to the morbidity and mortality after surgical intervention in neonates in LMIC’s. Tarek and colleagues suggest that up to 52% of neonates have some form of post-operative pneumonia, or bloodstream, surgical site or catheter-related infections.^5^ Early identification of post-operative sepsis and initiation of directed antimicrobial therapy can thus be both life-saving and economical, as it averts the protracted intensive care requirements of neonatal septic shock. To accomplish this, our high-volume Neonatal Intensive Care Unit (NICU) in Pakistan employs a standardized protocol of routine laboratory screening, patient isolation and prophylactic antimicrobial treatment in all post-operative patients with the goal of identifying and treating potential cases of neonatal sepsis before they reach a point of clinical decompensation.

While this protocol may seem intuitive, this is not evidence-based practice, and it is unclear what benefit, if any, this has had for recognizing post operative sepsis at our center. Recognizing this as an opportunity for quality improvement as well as assessment of practices, we aimed to conduct a clinical audit investigating the utility of post-operative laboratory screening for the identification of post-operative sepsis in an LMIC NICU setting. We hypothesized that the additional laboratory screening protocols would not result in an earlier diagnosis of neonatal sepsis, resulting in additional resource use without additional benefit. Furthermore, while inflammatory biomarkers such as C-Reactive Protein (CRP) have been investigated as screening tools for early onset neonatal sepsis, they have not been evaluated for screening in the immediate post-operative period. Therefore, as a secondary aim, we investigated the diagnostic validity of CRP testing for the screening of post-operative neonatal sepsis. The implications of this study reach beyond our single center, as they stress the importance of evaluating treatment protocols in LMIC NICUs to optimize patient outcomes while limiting unnecessary resource use.

## METHODOLOGY

We conducted a retrospective study of prevention and screening practices for post-operative sepsis at the NICU of our high-volume, specialized tertiary care center in Karachi, Pakistan. It is currently standard practice to test blood cultures and CRP levels on all post-operative patients on post-operative day (POD) 1, regardless of their clinical status, with the rationale of screening for post-operative sepsis. Furthermore, given the physical structure of our NICU (similar to other NICUs in Pakistan and other LMICs), patients are cared for in shared NICU wards, with multiple patients physically present in a single ward at any given point in time. Therefore, to prevent a potential spread of operating room pathogens, all post operative patients are put into “isolation” for up to 48 hours until they clear their first set of screening laboratory results.

### Ethical Approval:

It was obtained from the Ethics Committee (Ref. No.: 2023-8913-25274; dated June 14, 2023).

### Inclusion and Exclusion criteria:

For the purpose of this study, we included all neonates, defined as patients under 28 days of life or 44 weeks corrected gestational age, who underwent any abdominopelvic surgical procedure and were subsequently admitted to the NICU from 1^st^ January 2015 till 31^st^ December 2022. Relevant data on patient demographics, birth history, operative procedure, antimicrobial treatments, pre- and post-operative laboratory findings and culture results were collected retrospectively through patient medical records for descriptive analysis. Patients with incomplete or missing data, early discharge due to being transferred to a different hospital, those who left against medical advice or those undergoing neonatal cardiac or thoracic procedures were excluded (as these patients were managed in a dedicated cardiac ICU with separate treatment protocols). Finally, patients who did not receive any post-operative cultures (typically due to mortality prior to the first culture) were excluded as well.

### Data collection:

We assessed serial blood culture and CRP levels that were collected in all post-operative patients up to 14 days from the date of initial surgery. The test characteristics (sensitivity, specificity) for screening laboratory tests were determined by comparing their results against whether the patient developed true sepsis (gold standard). For this study, sepsis was defined as bacteremia with non-commensal pathogens on *any* post-operative blood cultures before POD 14, taking blood cultures as the gold standard for diagnosis of sepsis.^6^ In the absence of a standardized definition of neonatal post-operative sepsis, we did not consider clinical and physiologic parameters in our determination of which patients had post-operative sepsis – a common practice for the definition of sepsis in current literature and clinical trials.^7^ Test characteristics for blood culture and CRP screening were computed for each individual post-operative day to determine the optimal day for screening.

### Statistical analysis:

All statistical analysis was conducted using the R Program for Statistical Computing Version 4.4.2. Qualitative variables are presented as frequencies with percentages and quantitative variables are presented as means with standard deviation or medians with interquartile ranges. Post-operative CRP trends were represented graphically as scatterplots. Test characteristics (sensitivity, specificity) were calculated by constructing contingency tables of test positivity in reference to true sepsis for POD 0-2. Receiver operator characteristic curves were constructed to identify an optimal cutoff at which immediate post-operative (POD 0-2) CRP may predict neonatal sepsis.

## RESULTS

A total of 1757 patients admitted to the NICU at our center over the study period were screened for the purpose of this audit, among whom 109 patients underwent 115 surgical procedures. Overall, the median gestational age and birth weight of our patient cohort were 37 weeks (IQR 34 – 40) and 2.4kg (IQR: 2.0 – 2kg), and 33.9% were male (Table-I). All patients underwent some form of abdominal procedure, with necrotizing enterocolitis/spontaneous intestinal perforation being the most common indication for surgery (32.1%), followed by duodenal atresia (15.6%) and malrotation/midgut volvulus (11.9%). A total of 97 (94.2%) patients had a preoperative blood culture available, out of which 10 (9.7%) were positive. Post-operatively, 13 (12.6%) patients developed sepsis within 14 days of their surgery.

**Table-I:** Demographic and Clinical Characteristics of Patient Cohort.

Characteristic	Overall N=109
Age at the Time of Surgery (days)	5 (2 – 17)
Male sex	37 (33.9)
Gestational Age at Birth	37 (34 – 40)
Adjusted Gestational Age at Surgery	38 (37 – 40)
Birth Weight (kg)	2.4 (2.0 – 2.8)
Weight at Surgery (kg)	2.6 (2.3 – 2.9)
***Primary Diagnosis***	
NEC/SIP Duodenal Atresia	35 (32.1) 17 (15.6)
Malrotation/Volvulus	13 (11.9)
Anorectal Malformation	13 (11.9)
Jejunal/ileal atresia	11 (10.1)
***Postoperative Antibiotic Regimens***	
Cefotaxime	47 (43.1)
Amikacin	42 (38.5)
Meropenem	38 (34.9)
Vancomycin	25 (22.9)
Meropenem	25 (22.9)
Preoperative Culture Positive Sepsis	10 (9.7)
Postoperative Culture Positive Sepsis^2^	13 (12.6)
Postoperative CRP levels (overall)	6 (1 – 18)
Postoperative CRP levels (first 48 hours after surgery)	9 (2 – 36)

^1^Mean (%),

2 Median (IQR).

### Blood Culture Positivity by Post-operative Day:

**Fig.1:**
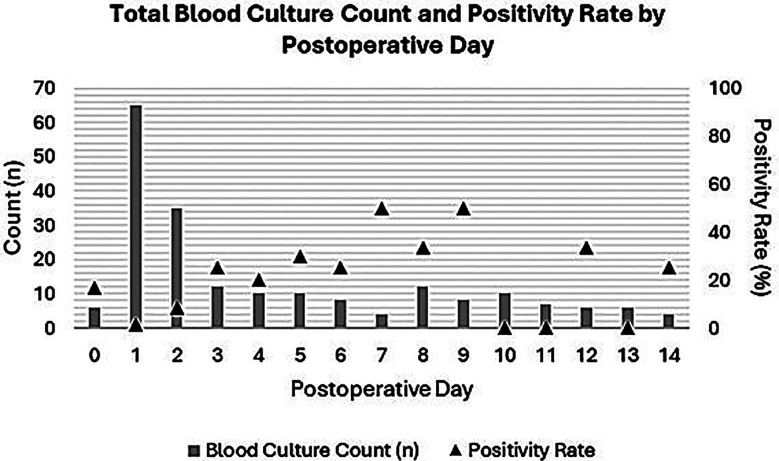
Blood culture utilization and positivity.

A total of 203 blood culture samples were taken from post-operative NICU patients within the first 14 days of surgery (Fig.1). Blood culture utilization was highest in the immediate post-operative period (POD 0-2), reflecting the screening protocol outlined earlier, with decreasing utilization in the subsequent 2-week period. Two patients had positive cultures within the first 48 hours after surgery, both of whom also had positive cultures immediately prior to surgery (indicating sepsis existing pre-operatively). New bacterial growth (i.e. growth of pathogenic organisms in post-operative cultures which were not present on preoperative cultures) was first noted on POD-2. Blood culture positivity rate, defined as the prevalence of positive cultures among all cultures performed on a given POD, increased steadily after POD-2.

### Sensitivity and Specificity of Post-operative CRP for Identifying Neonatal Sepsis:

**Fig.2:**
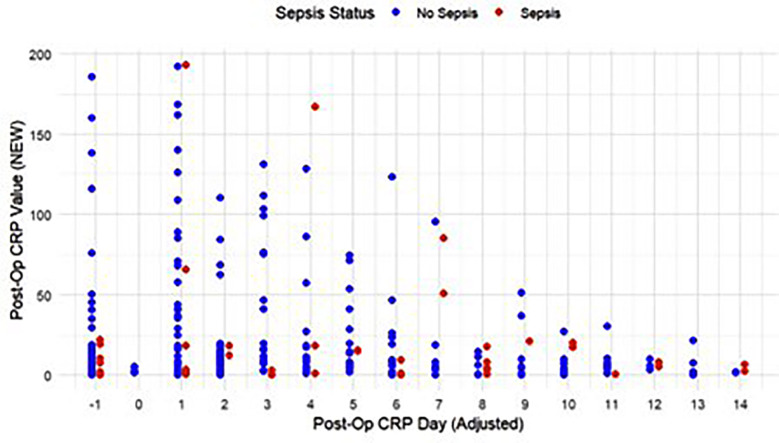
Trends in Postoperative CRP.

**Fig.3:**
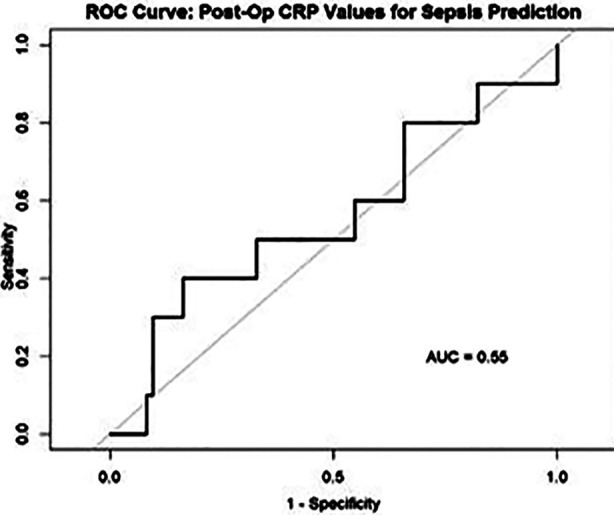
ROC curve for CRPs done on POD 0-2.

A total of 308 post-operative screening CRP tests were conducted, including 83 in the first two post-operative days. Similar to the observed trends of blood culture utilization, CRP screening was conducted most frequently in the immediate post-operative period (POD 0-2). Fig.2 summarizes all post-operative CRP screening values conducted for patients and graphically represents its discrimination between patients who developed and did not develop neonatal sepsis. Using a pre-defined CRP cutoff to predict sepsis (>10mg/L), the sensitivity (0.50), specificity (0.52), positive predictive value (0.52) and negative predictive values (0.88) were calculated. Receiver operator characteristic curves to test the utility of immediate post-operative CRP screening (within the first two post-operative days) found that initial post-operative CRP was unable to discriminate between cases of patients who developed post-operative sepsis versus those who did not (AUC = 0.55) (Fig.3).

## DISCUSSION

This clinical audit of laboratory screening practices for post-operative neonatal sepsis in an LMIC setting makes three important contributions to literature. First, we demonstrate that routine blood culture screening in the immediate post-operative period does not appear to correlate with presumed “true” culture-positive sepsis, likely due to the latency between initial bacterial exposure and subsequently developing bacteremia, or the fact that they simply did not have bacteremia. Similarly, our findings demonstrate that post-operative CRP screening demonstrated no discrimination between patients who developed sepsis versus those who did not. Finally, we highlight the value of conducting clinical audits in LMIC settings as a means to identify and limit areas of unnecessary or ineffectual resource utilization.

### The Utility of Post-operative Laboratory Screening in Neonates in LMICs:

Neonatal sepsis in the post-operative period is a highly heterogenous disease entity with multiple possible sources of microbial contamination. While post-operative neonatal sepsis is typically considered a form of late onset sepsis (characterized by nosocomial or horizontal pathogen acquisition from the operative environment), neonates undergoing surgery within the first few days of life are also susceptible to infections from vertically transmitted microbes. Furthermore, the relatively high prevalence of known risk factors for sepsis such as concomitant prematurity – particularly in neonates undergoing intestinal resection for necrotizing enterocolitis or spontaneous intestinal perforation – also adds to the challenges of diagnosing and appropriately managing post-operative sepsis. The recognition that post-operative neonates are at a higher risk of sepsis while also being more difficult to diagnose has prompted the investigation of an array of inflammatory biomarkers, including CRP and WBC, to screen for and diagnose sepsis in post-operative neonates.^8^

Our study demonstrated that CRP and WBC counts in the immediate postoperative period (POD 0-1) were poor predictors of post-operative sepsis. Given the innate, physiological inflammatory reaction to fresh surgical stress, a rise in acute phase reactants such as CRP is expected irrespective of the presence of an infectious source.^9^-^11^ Furthermore, leukocytosis with neutrophilia are typical components of a postoperative stress response and are known to peak at 24-48 hours after surgery in adults.^9^,^12^ As a result, these markers often fail to distinguish between non-infectious post-operative inflammation and early sepsis, as reiterated by our findings.^13^ Furthermore, we were unable to identify a higher diagnostic threshold to predict postoperative neonatal sepsis for either of these tests, suggesting that physiologic postoperative inflammation obscures any changes in CRP or WBC due to an infection or sepsis. Notably, CRP levels decreased with time, suggesting CRP levels on later postoperative days may have superior specificity for sepsis following neonatal surgery. This is in-line with literature in adults, which suggests that CRP is 94.4% sensitive on POD 4 and 88.8% specific on POD 6 as a screening tool for postoperative sepsis.^14^ A similar trend was seen with respect to postoperative blood cultures, which often failed to detect postoperative bacteremia in the immediate post-operative period in our study. Of note, positive blood cultures were predominantly observed after POD 2, suggesting that microorganisms inoculated during surgery took nearly 48 hours to reach detectable levels on culture.^15^

To an extent, these findings simply reiterate those of prior studies which have demonstrated overutilization of diagnostic testing in ICU settings with minimal clinical justification.^16^ However, while such non-invasive testing persists due to its perceived low-risk, low-morbidity nature, in the context of neonatal surgical care in LMICs they represent an unjustifiable source of additional resource and economic costs. In addition to the cost of testing (which itself may be unaffordable for low-income patients), false-positive cultures or falsely elevated inflammatory markers may trigger unnecessary clinical interventions, antibiotic use, patient isolation and prolonged length of stay, as was the case with neonates tested at our center. Such practices impose significant burdens on healthcare systems already constrained by resource limitations. Furthermore, in LMICs such as Pakistan with a lack of readily available health financing mechanisms or health insurance, patients and their families often have to pay out of pocket (OOP) for essential surgical care. In this setting, additional unnecessary testing and clinical interventions can translate into catastrophic health expenditures and financial toxicity, with long-lasting socioeconomic and health impacts for patients and their families.^4^,^17^-^19^

### Lessons on Clinical Audits in LMICs:

In high-income countries (HICs), established epidemiologic and quality improvement programs utilizing electronic health record (EHR) records or national registries such as the National Surgical Quality Improvement Program (NSQIP), make quality standardization and resource audits achievable and accessible. However, LMICs often lack the infrastructure for standardized data collection and centralized databases or EHRs, making QI initiatives challenging. Targeted, local clinical audits are thus an important alternative practice for improving patient quality and safety. Although labor-intensive and time-consuming, such audits can help in identifying problems in care, informing practices, and reducing uneconomical interventions.^20^ While audits are typically viewed as an intervention to improve patient care *quality* (such as audits to improve antibiotic or radiation stewardship) this study demonstrates the applications of clinical audits as a means to improve resource stewardship and reduce patient *costs*. Importantly, as demonstrated by this study, even simple, targeted audits can reveal ineffectual healthcare practices and opportunities for better resource stewardship in LMIC NICUs.

A lack of audit culture has been observed in LMICs despite their clear benefits, particularly in the setting of neonatal surgical and critical care.^21^ Multiple barriers hinder the implementation of effective audits in LMICs.^22^,^23^ These include inadequate resources, limited expertise in planning and analysis, teams working in silos, lack of an overall plan for audit, and logistic obstacles at organizational and/or national levels.^24^ The QuADRANT survey identified additional barriers to the utilization of clinical audits such as low national and hospital priority, lack of dedicated time, and most importantly a limited understanding of audit objectives among healthcare providers.^21^

Several strategies have previously been outlined to promote a sustainable culture of clinical audits, several of which are relevant to LMIC NICUs such as our own. These include structured audit training for staff, establishing dedicated staff for audits, allocating protected time to staff performing audits, and fostering a shared mental model between providers regarding goals of the audit.^21^-^24^ NICUs in resource limited settings may consider initiating or expanding resource audits to ensure care provision is cost-effective without compromising patient outcomes.

### Strengths and Limitations:

The principal limitation of this study was the single-center nature of its design, which may limit the generalizability and external validity of these findings. However, this further emphasizes the need hospitals and NICUs to conduct independent, internal audits to generate contextually relevant practice recommendations. Furthermore, given the retrospective nature of this audit, certain variables and data elements had extensive missing data. As a result, some cases had to be excluded due to missingness, which may have introduced inadvertent bias into our results. This again emphasizes the need to adopt audit culture into a hospital ecosystem, prospectively streamlining clinical data reporting for future anticipated audits. Nevertheless, this study addresses an important and poorly studied issue of overutilization of testing in surgical NICU settings, with potential implications for diagnostic stewardship and healthcare costs in LMICs.

## CONCLUSION

Clinical audits are valuable interventions to improve resource stewardship and optimize laboratory testing. Our clinical audit at an LMIC NICU demonstrated that early postoperative laboratory screening was not an effective screening tool for neonatal postoperative sepsis. By minimizing unnecessary testing and adopting evidence-based approaches, healthcare providers can reduce the out-of-pocket financial burden on families and optimize the utilization of scarce healthcare resources. Moreover, strengthening audit culture through capacity building and institutional support can serve as a catalyst for broader QI efforts across healthcare systems in LMICs.

### Authors Contribution

**HZ** conceived and designed the study, performed the statistical analysis, and drafted the manuscript, is responsible for integrity of research.

**RD and MOK** contributed to data analysis, interpretation, and manuscript drafting.

**JJ** reviewed and edited the final version of the manuscript.

**SS and ZI** were responsible for data collection.

**MAS, SQ, and FAK** provided supervision and critically reviewed the manuscript.

**SI** contributed to study conceptualization, provided supervision, and performed the final review of the manuscript.

All authors reviewed and approved the final manuscript and agree to be accountable for all aspects of the work.
